# UNICEF’s contribution to the adoption and implementation of option B+ for preventing mother-to-child transmission of HIV: a policy analysis

**DOI:** 10.1186/s12992-018-0369-2

**Published:** 2018-06-01

**Authors:** M. F. Chersich, E. Newbatt, K. Ng’oma, I. de Zoysa

**Affiliations:** 1 Wits Reproductive Health and HIV Institute, Faculty of Health Sciences, University of the Witwatersrand, Johannesburg, South Africa; 2 Itad Ltd, Hove, UK; 3 United Nations Children’s Fund, Pretoria, South Africa; 4 Development Horizons Ltd, London, UK

**Keywords:** HIV, UNICEF, PMTCT, Policy analysis, Option B+

## Abstract

**Background:**

Between 2011 and 2013, global and national guidelines for preventing mother-to-child transmission (PMTCT) of HIV shifted to recommend Option B+, the provision of lifelong antiretroviral treatment for all HIV-infected pregnant women.

**Methods:**

We aimed to analyse how Option B+ reached the policy agenda, and unpack the processes, actors and politics that explain its adoption, with a focus on examining UNICEF’s contribution to these events. Analysis drew on published articles and other documentation, 30 key informants interviews with staff at UNICEF, partner organisations and government officials, and country case studies. Cameroon, India, South Africa and Zimbabwe were each visited for 5–8 days. Interview transcripts were analysed using Dedoose software, reviewed several times and then coded thematically.

**Results:**

A national policy initiative in Malawi in 2011, in which the country adopted Option B+, rather than existing WHO recommended regimens, irrevocably placed the policy on the global agenda. UNICEF and other organisations recognised the policy’s potential impact and strategically crafted arguments to support it, framing these around operational considerations, cost-effectiveness and values. As ‘policy entrepreneurs’, these organisations vigorously promoted the policy through a variety of channels and means, overcoming concerted opposition. WHO, on the basis of scanty evidence, released a series of documents towards the policy’s endorsement, paving the way for its widespread adoption. National-level policy transformation was rapid and definitive, distinct from previous incremental policy processes. Many organisations, including UNICEF, facilitated these changes in country, acting individually, or in concert.

**Conclusions:**

The adoption of the Option B+ policy marked a departure from established processes for PMTCT policy formulation which had been led by WHO with the support of technical experts, and in which recommendations were developed following shifts in evidence. Rather, changes were spurred by a country-level initiative, and a set of strategically framed arguments that resonated with funders and country-level actors. This bottom-up approach, supported by normative agencies, was transformative. For UNICEF, alignment between the organisation’s country focus and the policy’s underpinning values, enabled it to work with partners and accelerate widespread policy change.

## Background

Programmes to prevent mother-to-child transmission of HIV (PMTCT) have undergone substantial transformation over the past ten years, both conceptually and in practice. The impact of PMTCT programmes has been dramatic, with some commentators describing it as “one of the great(est) public health achievements of recent times” [1, 2]. The expansion of PMTCT programmes accelerated after 2011, which saw the launch of the UNAIDS ‘Global Plan towards the elimination of new HIV infections among children by 2015 and keeping their mothers alive’ [1, 3]. Following the launch of the Global Plan, global coverage of antiretroviral (ARV) drugs for PMTCT increased markedly, from 50% in 2010 to 77% in 2015 [4]. More impressively, in the 22 countries with the highest burden of HIV, the proportion of HIV-infected pregnant women receiving lifelong antiretroviral treatment (ART) rose from 15 to 74% between 2010 and 2015 [1] and the number of new HIV infections among children declined by 51% over the same period [4]. Progress towards global targets, however, has varied considerably between countries, and over the past decade the field has seen a rapidly evolving evidence base, and frequent and often controversial policy changes [5–7].

By the late 1990s and early 2000s, high-income countries and some middle-income countries were providing triple-antiretroviral drug regimens to HIV-infected pregnant women, who were also strongly advised not to breastfeed their infants [8–11]. Among women who were not yet eligible for ART, the drugs were mostly discontinued after childbirth, but as early as 2001, the United States guidelines recommended considering lifelong continuation of treatment, regardless of CD4 count [12]. The countries that adopted triple regimens early on focused on optimising service coverage and have made very minor changes to policy thereafter [11]. In contrast, pilot sites for PMTCT in low- and middle-income countries emerged around 2000 and their expansion was initially very slow [13–15]. The early programmes were mostly built around single-dose nevirapine, with the shift to more effective regimens only occurring after the release of the 2004 WHO guidelines [16]. These guidelines were updated in 2006 [17] and again in 2010, when two alternatives, known as Option A and Option B, were presented (Table 1) [18]. In Option A, women are offered different combinations of drugs in pregnancy, childbirth and postpartum, and there are also varying regimens for infants, depending on infant feeding practices. In Option B, women receive triple-ARV prophylaxis from the third month of pregnancy until one week after cessation of breastfeeding. Countries were advised to choose from these regimens based on operational considerations [18]. With both Options A and B, initiation of ART was recommended for all women meeting ART eligibility criteria, including CD4 counts ≤350 cells/mm^3^. Option B+ emerged in 2011 as an alternative approach, in which all HIV-infected pregnant women would initiate lifelong ART, *regardless of CD4 count*.

**Table 1:** Description of Options A, B and B+ and level of evidence

Option	Year of WHO guideline	Regimen for woman		Regimen for infant	GRADE rating
		Treatment (CD4 ≤ 350)	Prophylaxis (CD4 > 350)		
Option A	2010^a^	ART	Pregnancy: AZT Labour: single-dose NVP & AZT/3TC Postpartum: AZT/3TC 7 days	NVP to 1 week after breastfeeding, or 4–6 weeks if not breastfeeding	Strong recommendation Low-quality evidence
Option B	2010 & 2013	ART	Pregnancy and labour: triple ARVs Postpartum until 1 week after breastfeeding	NVP or AZT for 4–6 weeks	2010: Strong recommendation moderate evidence 2013: Conditional recommendation Low-quality evidence
Option B+	2013	ART, regardless of CD4 count		NVP or AZT for 4–6 weeks	Conditional recommendation Low-quality evidence

^a^Option B+ included as a research priority in these guidelines

The role of different actors in guidelines formulation and enactment is important to understand. Among the global actors working on HIV, and within health more broadly, WHO has historically been charged with setting normative standards [19]. Generally, guideline revision processes are triggered by advances in evidence and, more recently, recommendations have been made on the basis of systematic reviews and the GRADE system that evaluates the quality of evidence [20, 21]. WHO and partners then disseminate guidelines in countries and assist with adapting the recommendations to national circumstances and supporting their implementation. These processes are lengthy (guideline revisions may take a year or more, and country adaptation and implementation even longer) and are seldom circumvented.

**Fig. 1:**
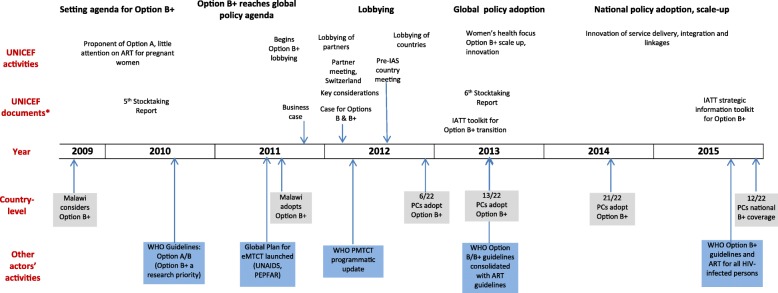
Chronology of Option B+ policy: steps in policy process and key events for UNICEF and other actors. *Most documents include other partners; with IATT documents, UNICEF is one of several partners. PC priority country, IAS International AIDS Society

While the influence of global organisations and other actors on policy making in the broader HIV field has been examined in detail [22, 23], less attention has been given to analysing their influence on PMTCT policy. Here, we examine the unique set of challenges, policy alternatives and political contexts that converged in 2010–2015 (Fig. 1), transforming the way that the PMTCT field itself was conceptualised. The study specifically assesses UNICEF’s contribution to this process.

## Methods

The study used a subset of data from an evaluation commissioned by UNICEF to examine aspects of the organisation’s work in PMTCT, and the paediatric HIV care and treatment programme in the period 2005-2015 [24]. Evaluation methods, fully described elsewhere [24], included document review, key informant interviews and country case studies.

### Document review

The documentation included global strategic planning documents and progress reports, as well as guidelines, policy and advocacy documents. Documents developed by international agencies were obtained by searching the websites of UNAIDS, UNICEF and WHO, and the Inter-Agency Task Team (IATT) for Prevention and Treatment of HIV Infection in Pregnant Women, Mothers and Children [25, 26]. Journal articles were located through a search of Medline (Pubmed) done in January 2017 using the free text search terms ((Option B+ OR Option B Plus) AND HIV). Additional papers were located using more targeted searches of Medline, as required.

### Key informant interviews and country case studies

Potential interviewees were identified initially through a process of consultation with UNICEF, and supplemented with additional respondents on the basis of the evaluation team’s collective knowledge of the HIV/AIDS sector. The aim was to achieve a balance of perspectives between UNICEF, partners and government officials.

Interviewers followed a structured interview guide containing open-ended questions covering a range of topics, including factors influencing changes in PMTCT policies. They were carried out by six members of the evaluation team, in person or by phone. Prior to each interview, we explained the purpose of the discussion and measures to protect interviewees’ confidentiality and anonymity. We also confirmed that they had understood the purpose of the interview and agreed to audio record the discussion. Interviewees were specifically informed that participation without being recorded was possible, in which case detailed notes were taken by the interviewer and later transcribed (a few respondents chose this option). All other interviews were fully transcribed. The study procedures were reviewed and approved by the UNICEF Evaluation Office in UNICEF Head Quarters in New York.

In total, the evaluation entailed 243 interviews. In a subset of interviews (*n* = 30) respondents were specifically asked about their experiences with the adoption and implementation of Option B+. These interviews were included in this sub-analysis. At global and regional levels, the full evaluation included interviews with UNICEF regional and headquarters staff (*n* = 44); and representatives from the Global Fund to Fight AIDS, Tuberculosis and Malaria (the Global Fund; *n* = 2), UNAIDS (*n* = 4); UNFPA (*n* = 2), WHO (*n* = 5); and other international and national non-government organisations. Respondents at country level consisted of 48 government officials, 61 UNICEF country office staff, and 84 individuals from development partners, civil society organisations and other relevant actors. To protect confidentiality, we do not use respondent’s names, but instead classify them into four broad categories, specifically, ‘UNICEF staff’, ‘UN partner organisation’, or ‘other development partner’, regardless of whether they worked at headquarters, regional or country level; and ‘respondents at country level’, which includes both government and other national stakeholders. Field visits, encompassing interviews and a desk review, lasted 5–8 days and were made to 4 of the 22 Global Plan priority countries (Cameroon, India, South Africa and Zimbabwe). Data from the country case studies were integrated into the narrative of the final report.

### Data analysis

Using Dedoose software [27], relevant text was extracted from interview transcripts and set aside for more detailed analysis. The transcripts were also fully reviewed, in order to ground the extracted data within the context of the interview as a whole. A single reviewer (MFC) coded interview extracts according to the emergent themes that best summarised and explained the evolution of policies and programmes to Option B+. Two other reviewers (IdZ and EN) then cross-checked the coding and differences in interpretation were resolved through discussion. The themes then formed an overarching framework, which was supplemented by the findings of the document review.

In the first section of the article, we apply the Kingdon multiple-streams theory to examine how the Option B+ policy emerged. This analysis considers three ‘streams’: the ‘problem’ or deficiencies with the existing policies (Option A/B); the policy alternative that emerged in response to these deficiencies (Option B+); and relevant political factors at the time. We document how the convergence of these streams pushed Option B+ onto the policy agenda and thus compelled policy makers to make a decision about its adoption [28, 29].

Thereafter, the ‘policy triangle framework’ [30, 31] is used to understand how political contexts influenced the direction and feasibility of policy-making at global and then country level. We aim to draw out the influence and interactions of various actors – specifically focusing on UNICEF – to map the process trajectories and identify how these shaped the policy content. Finally, within the political context in the period under review, we note what the actions of UNICEF tell us about its character as an organisation. Illustrative quotes from participants are provided, where relevant.

## Results

### Option B+ reaches the policy agenda

**Fig. 2:**
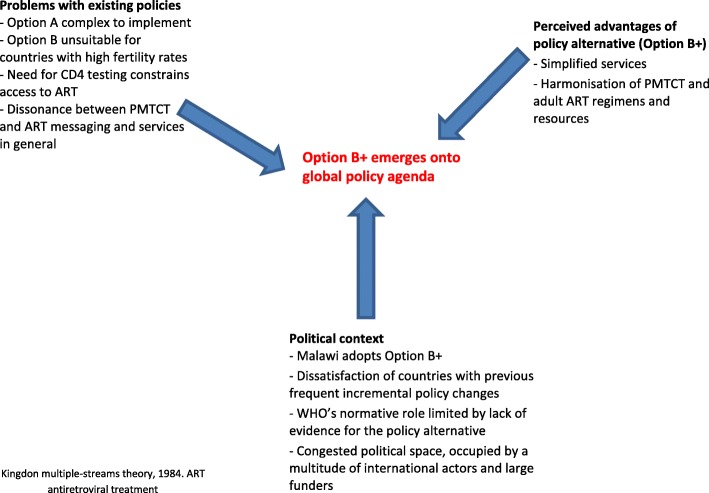
Option B+ reaches the global policy agenda: a confluence of three streams, problems with existing policy, a compelling policy alternative and a conducive political context. Kingdon multiple-streams theory, 1984

By 2010, it was clear that most high-burden countries were facing considerable challenges in scaling up services built around the PMTCT regimens recommended at the time (Fig. 2) [6, 32]. Services were limited by gaps in HIV testing and the substantial drop-off of women in each of the sequential steps that constitute the ‘PMTCT cascade’ [33, 34]. Further, the drug regimens in Option A and B, articulated in the WHO 2010 guidelines, were even more complex than their predecessors as they included ARVs to prevent HIV transmission during breastfeeding. Option A/B also required CD4 testing to identify pregnant women eligible for ART (Table 1), which was challenging in many primary care settings [35, 36], especially in rural areas [37–39].

In late 2009, even before the WHO 2010 guidelines were formally launched, the Malawi government and its development partners had begun to consider the Option B+ policy [40]. The simplicity of using only one ARV regimen for all pregnant women, and not having to perform a CD4 count on pregnant women had much appeal. Two respondents in our study noted that the Malawian national technical working group, formed by merging the PMTCT and ART working groups to encourage linkages between these areas, was concerned that Option A was too complex to implement locally [41]. In addition, they felt that Option B (which required starting ART, but stopping when breastfeeding ceased) would not be practical in a setting where intervals between pregnancies were typically short [41]. A country-level respondent recalled that the view was that: “*Option B was simple on paper, but not so simple in practice”.* At the time Malawi faced considerable weaknesses in the systems needed to support PMTCT and ART programming, and thus the guidelines had to be simple enough to be implemented at the smallest and most remote health facilities and by lower level health cadres [42]. Among other concerns, only about 50 of the 417 ART clinics in the country had a working CD4 machine, meaning that the majority of pregnant women would not be assessed for ART eligibility [43, 44]. Also, there were concerning reports of high mortality among HIV-infected women during the postpartum period, even among those with a CD4 count above 350 [45], which suggested to the working group that treatment for life would likely be beneficial for all HIV-infected pregnant women.

In short, the more apparent the challenges in implementing the existing policies for Option A/B became, the more compelling was the case for a competing alternative: the time was ripe for a major shift in global policy. Importantly, with Option B+, the same ARV regimen would be used for PMTCT and for adult ART. According to one respondent working at country level in Malawi, this simplification was, in fact, the most compelling argument for Option B. Malawi’s position in this regard was foreshadowed by their long-standing resistance to using laboratory technology such as CD4 counts to support its ART services, preferring instead simplified clinical and programmatic approaches [46].

After developing a set of tools, including a guideline that integrated PMTCT and ART recommendations [40, 47], the Malawian Ministry of Health formally adopted the Option B+ policy in mid-2011. In doing so, the Ministry pre-empted the usual approach of waiting for WHO to absorb emerging data on new interventions and release updated guidelines. This circumvention, however, meant that an application by the Ministry of Health for support from the Global Fund to Fight AIDS, Tuberculosis and Malaria for its scale-up plan in 2011 was initially unsuccessful, given that the Global Fund could only support interventions that were consistent with WHO recommendations.

Under the coordination of the Malawi Technical Working Group and with financial resources from a revision of the existing Global Fund grant budgets and considerable new funding from PEPFAR and other agencies [40, 42], Option B+ was rapidly rolled out across all health facilities in Malawi between July and December 2011, which is a remarkable achievement. Several partners, including the UNICEF country office, played a key role in this process. A UN partner agency representative noted that “*UNICEF was a lead in terms of supporting Malawi* [in implementation of Option B+]”. Though UNICEF had earlier been a firm proponent of Option A, the country office rallied to support the efforts of the Malawi Ministry of Health around Option B+. According to a UNICEF staff member, this support took the form of facilitating and funding key meetings, helping to develop guidelines and planning tools, training health workers in Option B+, and assisting with the estimation and management of commodities for HIV. Meanwhile, other countries, for example, Zimbabwe and Haiti, also began voicing similar concerns about Option A/B and considering a shift to Option B+ [36, 48].

### Option B+ becomes global policy

**Fig. 3:**
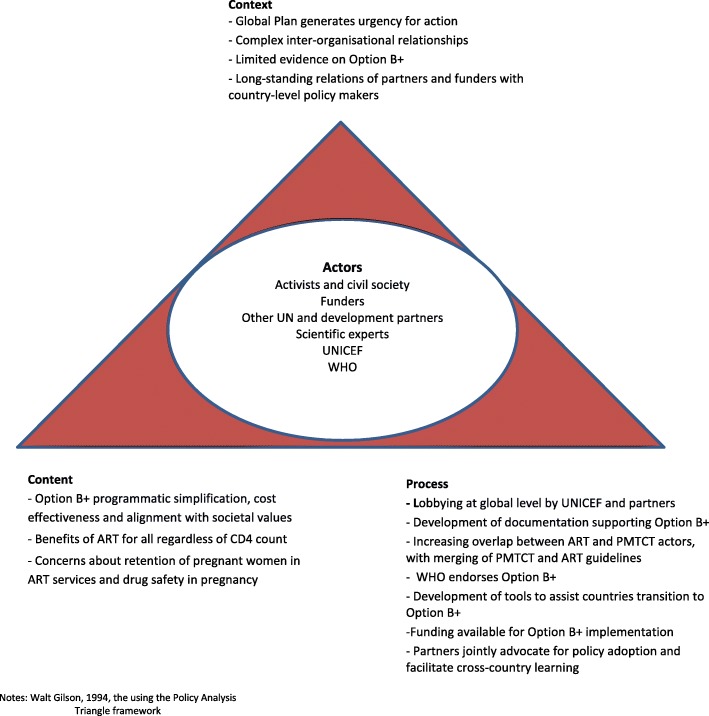
Adoption of Option B+ as global and national policy: an analysis of context, process and content using the Policy Analysis Triangle framework. Walt Gilson, 1994

Malawi’s bold policy initiative unfolded within the context of a PMTCT field that was ready for a new approach, one that better reflected the programmatic realities in most countries and would mark a departure from the incremental, somewhat confusing, policy changes of the preceding decade (Fig. 3) [49]. A review of PMTCT policy shifts in Tanzania noted that the frequency of change, in itself, had generated a desire for a new approach: “*The continuously changing recommendations on PMTCT stress the need for a much simpler and [more] effective approach*” [39].

#### UNICEF and partners back Option B+, and lobby partners and country-level actors

After a period of hesitation, staff in UNICEF headquarters, including at its most senior level, recognised the potential of Option B+ to overcome the programmatic challenges posed by Options A/B. In the years preceding Option B+, UNICEF documentation did not raise the concerns about Option A/B that were highlighted so clearly in their arguments for Option B+ thereafter [50, 51]. In fact, evaluation respondents have drawn attention to the fact that UNICEF had tended to favour Option A, which formed the basis for the ARV drugs included in the Mother-Baby pack that the organisation had been promoting in some countries in 2010 and 2011 [51]. Then in early 2012, spurred on by promising findings of a cost-effectiveness analysis and a deepening recognition of the potential advantages of Option B+, the policy began to appear prominently in documentation produced by UNICEF and partners [52–54].

UNICEF staff and others described how UNICEF then became a vigorous and vocal proponent of a transition to Option B+. The policy, according to a UNICEF staff member, represented “*something that UNICEF could really grab onto and push”*. Support for Option B+ was also strongly reinforced in meetings of the IATT, which is co-convened by UNICEF and WHO. The IATT includes more than 30 partners and coordinates the provision of technical support and other activities of partners working on PMTCT. Momentum around the policy was also generated by a high-level advocacy meeting hosted by UNICEF and CHAI in Geneva, Switzerland, to discuss the policy with UNAIDS, WHO, PEPFAR, the Global Fund, EGPAF and other partners. Important technical discussions also took place among IATT partners in preparation for the release of the WHO programmatic update in April 2012.

UNICEF and other partners developed several documents proposing, in quite compelling language, the advantages and long-term cost savings of Option B+ [53, 54] and used these as the basis for their advocacy. They actively championed the Option B+ policy among partners, at ministerial level and at key international meetings [55]. For example, UNICEF played a pivotal role in coordinating events around the Option B+ policy at the May 2012 World Health Assembly and the July 2012 International AIDS Society Conference in Washington, which involved ministers of health and key partners from many countries who pledged support for Option B+.

#### Strategic framing of the policy

One UNICEF respondent recalled the mood generated by discussions of the Option B+ policy at the symposium preceding the Washington conference: “*I remember the excitement at the meeting in Washington in July 2012... Of course they grabbed onto immediately the simplicity of it from the health workers’ and the health system’s point of view”*. In addition to presenting these arguments, UNICEF framed its case for Option B+ around the ethical obligation to “*put the mother at the centre of this drive to eliminate vertical transmission, and not the child”* (quote from UNICEF respondent). This notion was the first item listed among the strategies to accelerate PMTCT in the UNICEF 2013 Stocktaking report [56]. A UNICEF respondent recalled that placing “*women first, not the child*” had demarcated a major shift from previous approaches to PMTCT, in which: “*it was all about children at the beginning”*. Also, importantly, the ambitious targets in the Global Plan [6] provided a platform for claims of urgency to be made, and for pressing countries to do things differently. As noted by a UNICEF respondent, the organisation and its partners drew on this, successfully arguing that Option B+ provided the best means of “*urgently getting things done”.* In addition, a the growing body of evidence pointed to the benefits of early initiation of ART in all HIV-infected persons [57], signalling an increasing convergence of ART and PMTCT programming, and the growing influence of ART experts in the PMTCT policy arena.

#### Opposition from other actors

The support of UNICEF and a few partner organisations for Option B+ [58] did not mean the path to global adoption was smooth: as one UNICEF respondent put it, the policy “*didn’t fly out”*. Another recalled that “*there was a lot of resistance to this from many quarters”*. These events were unfolding within a particularly congested political space, occupied by a multitude of international actors and large funders, and a very vocal group of seasoned scientific experts, who had a long history of engagement with policy making in PMTCT and often held polarised views [5]. International organisations and other actors were concerned about the possibility that retention rates of pregnant women on treatment would be poor, especially as there was much less capacity to follow-up pregnant women than there was for adults or children taking ART [59]. In other words, “*people were worried about putting women on treatment too early or so early that they wouldn’t be supported to be retained on treatment”* (UNICEF respondent). But, there were also concerns about the costs involved, especially of the additional drugs and that finances for an ambitious service like Option B+ would siphon already scarce resources away from other initiatives, or from improving already burdened health systems [60–62].

While Option B+ did attract support from some members of the scientific community [41, 44], several experts called for more detailed evaluation of the policy prior to its adoption [63, 64] and raised concerns about safety of the medication in pregnancy [5, 60, 61, 63, 65]. These concerns centred on the potential teratogenicity of efavirenz, which was used in adult ART regimens [63]. Over time, however, a large body of evidence was built up supporting the safety of the drug in pregnancy [63]. Commentators also critiqued the speed of policy change, noting that this had allowed for too little consultation and debate, and was characterised by the “*single-mindedness of advocacy”* [61]. Some even referred to the success of the Option B+ policy as “*a triumph of marketing over data”* [66]. The following quote perhaps best sums the views of some in the scientific community: *“…guidelines on preventing mother to child transmission of HIV have dangerously shifted from recommendations supported by strong scientific evidence, to recommendations based on experts’ best guesses and extrapolations followed by field evaluation, and then to recommendations [for Option B+] proposed on the ground of theoretical modelling and ideological principles, with limited possibilities for validation or refutation”* [5].

Many experts, together with several global policy makers, were also concerned about the lack of parity between ART for pregnant women and ART for other adults, where eligibility in the latter was still being determined by CD4 thresholds. This dissonance, they argued, implicitly prioritises treatment for relatively healthy pregnant women over that for sicker populations [5, 61, 67]. Lastly, only one respondent mentioned the role of HIV activists and civil society, noting that these groups had expressed strong reservations about starting ART for all HIV-infected pregnant women, owing to insufficient evidence, and as they feared women might be “coerced” onto treatment [68].

#### Final processes of policy adoption at global level

Notwithstanding its detractors, a critical mass of international and national actors eventually came to support the policy proposal and “*there was clearly a crescendo of interest and support”*, as recounted by one UN partner informant. Much of this support came from “*the high, high level leaders [who] really pushed in a positive direction and sometimes without a full scientific or cost based [assessment]”* (respondent from partner agency). As mentioned above, WHO convened technical meetings, towards the release of a programmatic update in April 2012, which proposed Option B+ in addition to Option B, and emphasized the potential advantages of these approaches over Option A [69]. The document noted that while Option B+ had important advantages over other options, especially Option A, it still needed to be evaluated in programme and field settings. Importantly, no peer-reviewed evidence was available at the time to support assertions about the potential advantages of Option B+.

Option B+ was then formally adopted by WHO in June 2013. The guidelines noted that Option B+ was recommended “for operational and programmatic reasons, particularly in generalized epidemics” and in recognition that, by that time, 40% of LMICs assessed in a survey of 112 countries had, in fact, already adopted Option B+ [70]. The only evidence cited that directly related to the effectiveness of the policy was a before-and-after study in Malawi based on programme-level data (Table 2). The data showed a remarkable increase in number of pregnant women started on ART with Option B+, but provided limited evidence on whether women were retained in care long-term and contained no biological outcomes [36, 71]. Despite the groundswell of support for the new regimen, however, the 2013 guidelines retained Option B alongside the newly-introduced Option B+, suggesting some reticence remained about Option B+ among global partners and the scientific community [5]. Moreover, for the first time, WHO presented a single guideline encompassing recommendations on both PMTCT and ART. This change, and the Option B+ policy as a whole, represented, according to a respondent from a UN partner, “*kind of the end of PMTCT if you will*”, with “*pregnant women just rolled up as just one sub-population within the aggregate numbers of people on ART*”. Others have similarly described this as the “complete integration of ART and PMTCT into a single program at each level of the health sector” [42], while one respondent viewed this more narrowly, as primarily being about a convergence between prevention of vertical transmission and ART, with PMTCT still retained as a distinct programme area with unique features.

**Table 2:** Available evidence on Option B+, by year

Year	Description of study	Description of study	Data on Option B+ effectiveness and costs
2011	Country-level report [87]	Quarterly summaries of PMTCT programme data in Malawi	Change in coverage of PMTCT and ART services for pregnant women in Malawi following introduction of Option B+
2012	Country-level reports [87]	Quarterly summaries of PMTCT programme data in Malawi	Further data on change in coverage of PMTCT and ART services for pregnant women in Malawi following introduction of Option B+
	International organisations document [54]	Cost effectiveness analysis (Business case)	Estimates of effectiveness and costs of Option B+ based on assumptions, not data
	Journal article [88]	Cost effectiveness modelling of individual countries	Estimates of effectiveness and costs of Option B+ based on assumptions, not data
2013	Evaluation report [76]	Evaluation of PMTCT programmes in Lesotho, Malawi, Tanzania and Zambia (10 facilities per country)	Comparison of ART coverage in HIV-infected pregnant women and PMTCT cascade assessment, comparing Option B+ programme in Malawi with other countries
	Journal article [36]	Before and after study	Number of pregnant women starting ART in Malawi and proportion receiving ART at 12 months, before and after Option B+
	Journal articles [89, 90]	Cost effectiveness modelling for individual countries	Estimates of effectiveness and costs of Option B+ based on assumptions, not data
2014	Journal articles [91, 92]	Retention of pregnant women in ART long-term	Proportion of pregnant women who initiate ART in Option B+ services retained in care
	Evaluation report [40]	Programme evaluation	Trends over time in PMTCT programme effectiveness in Malawi and barriers to Option B+
	Journal articles [93, 94]	Cost-effectiveness modelling of individual countries[9] and across regions	Estimates of effectiveness and costs of Option B+ based on assumptions, not data
2015	Journal articles [95, 96]	Effectiveness of Option B+ programmes for women and children	Proportion of pregnant women who initiate ART in Option B+ services retained in care and MTCT rates
	Journal article [76]	Costs of switching from Option B to Option B+	Estimates of effectiveness and costs of Option B+ based on assumptions, not data
	Journal article [97]	Randomised controlled trial	Demonstrated benefits of starting ART in all HIV-infected adults, regardless of CD4 count

### Country-level adoption and implementation of Option B+

#### Policy processes and interactions between international actors and countries

Against the background of the WHO 2012 programmatic update [72], in early 2013, IATT partners, including UNICEF [26], moved swiftly to develop a full suite of tools to support countries in policy costing, transition planning and then implementation of Option B+ [26, 73]. As activities of IATT are mostly done jointly by partners, it is difficult to disentangle the individual contribution of each agency. One respondent from a partner organisation summed this well: “*because so many people are working in this space with PMTCT and B+ roll out, it’s hard to partition off what was UNICEF, what was UNAIDS, what was WHO, what was PEPFAR”*. Nevertheless, some specific contributions were noted, with, for example, one respondent from a development partner remarking that: “*UNICEF really picked up the ball in their area of expertise, which in this particular instance was creating lots of tactical tools for countries to use”*. Several interviewees and a publication also noted that the technical tools designed by PEPFAR to support implementation of Option B+ had been especially useful [26]. Importantly, once the shift to Option B+ policy had been formalised at a global level, substantial PEPFAR, Global Fund and other financing quickly became available, further incentivising and supporting the policy’s adoption.

Though much of UNICEF’s technical assistance to countries was done in collaboration with partners, especially WHO, one respondent in the country-case studies reported that UNICEF country teams “*led the charge”* within many countries. Respondents in several countries pointed out that these activities were set within long-standing trust relations with health ministries, which made UNICEF an “*ideal organisation for Option B+, with countries poised for change”*. These respondents also noted that UNICEF’s strategy of gathering operational evidence and sharing experiences with Option B+ between countries had helped reassure governments in ‘late adopter’ countries that Option B+ was a viable alternative. This was done by arranging visits of health ministries to countries that had already implemented Option B+, setting up meetings involving policy makers from several countries and doing cross-country evaluations [74–77]. WHO organised large guideline dissemination meetings in all regions, and PEPFAR and other organisations led many country-level activities, often coordinated by the IATT.

UNICEF frequently worked in collaboration with other organisations to advocate for Option B+ within countries, in initiatives described as: “*We carried out joint visits to countries with other agencies from the system to push for a specific policy…. this brought about changes for the policies in many countries”*. Some scientific experts, however, viewed these efforts to ‘push’ the policy in a negative light, stating in one commentary that “*international agencies should guide, but not pressure, ministries into making decisions, particularly when evidence is weak”* [61]. While UNICEF clearly advocated for Option B+, it also supported countries in weighing up the operational and cost implications of the policy prior to its adoption. A UNICEF respondent reported that in Mozambique, for example, the Ministry of Health had been advised to *“make sure that if you go ahead in moving to B+, you know what you are doing and you can manage the additional amount [of patients receiving ART]*”.

#### Countries adopt and implement Option B+

The shift to Option B+ took place within a few years, a process that one UNICEF employee described as “*phenomenal […] in terms of the normal pace for countries of uptake of new WHO guidelines*”. By as little as one year after the 2013 WHO guidelines, 21 l of 22 Global Plan priority countries had adopted the policy. This was significantly faster than with previous guidelines. For example, a review of 70 countries in 2012 found that by then, less than half had adopted the WHO 2010 PMTCT guidelines and only 60% had taken up the adult ART recommendations [78]. And before that, uptake of the 2006 guidelines had been even slower: by 2009, 30% of HIV-infected pregnant women in the world were still receiving single-dose nevirapine regimens for PMTCT, contrary to recommendations in the 2006 WHO guidelines [79].

By the end of 2015, 12 of the 22 priority countries had achieved – or were close to achieving – national coverage of Option B+ services [1]. In many countries, the programmatic transition away from previous regimens was remarkably rapid [44]. In Zimbabwe, for example, on the back of a carefully designed operational plan and considerable financial and technical support from implementation partners, roll out was completed within a year [80, 81].

### The political context within which UNICEF operated

UNICEF’s active role in this chapter of PMTCT policy history had positive spin-offs for the organisation itself. Through their lobbying and technical leadership in promoting Option B+, UNICEF “*rediscovered its niche that had been lost in the preceding year or two”*, according to a partner organisation representative. The Global Plan initiative, led by UNAIDS and PEPFAR [58], had altered the balance of power between international partners working on PMTCT, with each partner having again to assert its position and define “*exactly [what] its niche would be within the Global Plan”* (respondent from UNICEF and partner organisation).

A UNICEF representative explained that, in addition to Option B+ having “*positioned us again”*, the activities around the policy had reinvigorated the organisation: “*it gave people a lot of motivation”*. Then, “*after the advocacy battle was over and the evidence had come in”*, UNICEF resumed its “*efforts to support countries to implement”* (UNICEF respondent).

Lastly, when examining UNICEF’s activities over the period, some features of the organisation stood out. Firstly, many respondents, both from UNICEF and beyond, remarked on UNICEF’s willingness and ability to take forward concepts and lobby at a high level, but also within strategic partnership for ideas that challenge conventional thinking. Doing so demonstrated a willingness to take risks, risks that might generate opposition, even antagonism among partners. A staff member described UNICEF’s promoting of Option B+ as an act that: “*required some courage”*. Secondly, a sense of urgency to get the job done appears to have been part of UNICEF’s ‘culture’, although this at times had led the organisation to “*run too quickly with ideas”* before they had been fully tested (UNICEF respondent). Thirdly, many of the principles underpinning Option B+ resonated with the core strengths of UNICEF and with the needs of countries’ PMTCT programmes at the time. According to UN partners and its own staff, UNICEF has traditionally been adept at championing and supporting interventions that are: technically feasible; operationally simplified; coherent with existing supply chains; easy to explain and translate at country level; and amenable to being framed around dominant societal values – in this instance, the health of mothers. It was this alignment between the organisation’s strengths, the needs of country-level actors at the time and the specific content of the Option B+ policy that enabled the organisation, in tandem with other partners, to take a lead in making the policy a reality.

## Discussion

Through concerted high-level lobbying, and UNICEF’s investment in developing the initial tools used to advocate for Option B+, provision of country-level technical assistance on implementation and then review of the early operational evidence around Option B+, the organisation made an important contribution to the transformation of PMTCT policies that took place. Within a complex political context, UNICEF – in unison with other actors – successfully crafted and propagated a compelling frame around the Option B+ policy, and in the process overcame substantial reservations around the policy, especially a relative lack of evidence to support it. UNICEF’s values and pragmatism synchronised with the underpinnings of Option B+, and their engagement with the policy allowed them to regain prominence within a congested global policy space. The organisation, together with partners such as PEPFAR, WHO and the IATT more broadly, had served as ‘policy entrepreneurs’: the actors who take the lead in promoting a policy through a variety of channels and means, and ultimately are crucial to its success [31].

A theme cutting through our findings is a story of how the normal top-down processes of global policy development were circumvented, driven instead by a national-level policy initiative in Malawi [46]. Typically, as in the PMTCT policy iterations prior to Option B+, biomedical evidence occupies centre stage in the formulation of new policy at an international level. Previous policy changes for PMTCT were mostly characterised by gradual or incremental changes, and relatively ‘low’ politics. The processes surrounding the ascendance of Option B+ were, however, clearly quite different. In fact, at the time of its formal adoption in 2013, Option B+ was given a GRADE review rating of ‘low-quality evidence’ and labelled a ‘conditional recommendation’, where the desirable effects of the recommendation only *probably* outweigh the undesirable effects [19]. The framing of the policy around principles and pragmatism, rather than evidence, had favoured the more substantive transformation or ‘shift’ in policy, which, in turn, generated a period of contentious ‘high’ politics. The near absence of evidence for Option B+ posed challenges for WHO in navigating the process of guideline development for Option B+. On the one hand WHO risked attracting criticism for making policy on the basis of little evidence [60, 61], while on the hand other the organisation needed to respond to the momentum gathering around the policy [82], and a growing number of countries planning to adopt Option B+, regardless of whether it was global policy, or not.

Overall, at a country level, it is apparent that many international actors and donors, including UNICEF had considerable influence, stemming from well-established relationships built around areas such as programmatic support, the perceived legitimacy of their technical advice and the receipt of considerable funding linked to the policies they promulgate.

Of note, the shift to Option B+ has had far-reaching consequences. Countries were able to focus on operational issues like patient retention [83], rather than on complex debates around which regimen to choose and how then to operationalise that decision. Also, the experiences with Option B+ served as a ‘proof of concept’ for the policy endorsed by WHO in 2015 of providing ART for life for all HIV-infected individuals [84, 85]. The convergence of PMTCT and ART programming, brought about through Option B+ and the entry of actors from the ART arena into the PMTCT policy space may, however, lead over time to the reduced visibility of PMTCT and diminished resources for the field.

### Study limitations

Given that we examined events spanning several years, there is a risk of recall bias, or of loss of institutional memory. To mitigate this, we included people who were active at different time points, and even those no longer working in the sector. Also, although our sample included interviewees across several stakeholder groups, we were unable to interview people from all relevant organisations. Further, respondents were often reflecting on their own work, or that of co-workers, a position that may have made it difficult to draw an objective assessment. Similarly, since some of the research team were ‘policy insiders’ who had worked in the PMTCT field, this status may have compromised our ability to be ‘neutral’ observers – although one’s positioning as insider or outsider vis-à-vis the particular group under study is more complex than this binary would suggest [86].

Lastly, and perhaps most importantly, this study was based on an evaluation which was commissioned by UNICEF; therefore potentially undermining the independence of the evaluation team. Several steps were taken to counter this possibility. Firstly, the evaluation team, rather than UNICEF staff, was responsible for the evaluation’s design and conduct, which was mediated through the UNICEF Evaluation Office, rather than the HIV section. Moreover, an extensive document review was done to corroborate the findings of the evaluation.

## Conclusion

In conclusion, Option B+ represented a ‘game changer’ for PMTCT [1]. The preceding period of incremental policy change was disrupted or punctuated by a burst of rapid policy transformation, stemming from a new understanding of the ‘PMTCT problem’ and a policy alternative that promised to overcome shortcomings of previous policies. All this was given considerable momentum by Malawi’s national-level policy initiative and the development of a set of strategically framed arguments, articulated within a field poised for innovation. UNICEF served as one of the leading agenda-setting agencies for Option B+ and as a key actor in securing its widespread, rapid implementation.
